# Supplementary material for: Evaluation of a novel biplex rapid diagnostic test for antibody responses to Loa loa and Onchocerca volvulus infections
Source: PLoS Negl Trop Dis. 2024 Oct 10;18(10):e0012567. doi: 10.1371/journal.pntd.0012567 (PMC11495599; doi:10.1371/journal.pntd.0012567)
Supplement: Attachment — Submitted filename: Revision Letter_Plos-TDR.docx [file pntd.0012567.s001.docx]

September the 3^rd^, 2024

Section Editor, PLOS Neglected Tropical Diseases

Dear Jong-Yil Chai,

We hereby submit the revised version of our manuscript entitled “**Evaluation of a novel biplex rapid diagnostic test for antibody responses to *Loa loa* and *Onchocerca volvulus* infections**” (PNTD-D-24-00589). We thank the three reviewers and the executive editor for their positive comments and appreciation of the paper.

We addressed each of the comments with detailed responses. We hope our manuscript will now meet the standards for publication.

Sincerely yours,

Jérémy CAMPILLO

jeremy.campillo@ird.fr

911 avenue Agropolis, 34000, Montpellier, France

*Tel: +33 4 67 41 61 62*

_________________________________________________________________________________**Reviewer #1**.

*This a very interesting manuscript on a new diagnostic test for field application on a difficult disease. The text is well written and reads well. There are however some very minor comments.*

- *Page 7, line 46: “discordance […] were investigated” please add a “s” at discordance.*
- *Page 17, line 223-224: some definition of the one step error (or more) would be helpful to the reader that is not familiar with validation processes*
- *Page 19, table 3: number of participants (N=) for each discordance model could be added for transparency*

**We thank reviewer 1 for his/her comments and overall appreciation of the article. We have:**

- **added the “s” to “discordance”.**
- **added an explanation for one-step errors (and more): “**This calculation assumed that a one-step error (a difference of one category between both ARTs, such as “1” vs. “2”) was equivalent to a two (“1” vs. “3” or “0” vs. “2”) or three-step error (“0” vs. “3”)**”.**
- **Added the number of participants for each discordance model (Table 3).**
- *Page 22, lines 290-295: wouldn’t it be possible to define a reference standard with IgG testing complemented with clinical manifestations and compare the IgG4 based test to get a more true sensitivity and specificity?*

**Indeed, the complexity of comparing diagnostic tests in loiasis is that there is no gold standard. In this article, we have focused on the evaluation of the IgG4 test in real-life, in field conditions without the possibility of performing IgG assays. Another article will be devoted to its laboratory evaluation on plasmas.**

________________________________________________________________________________

**Reviewer #2**.

- Are the conclusions supported by the data presented? Yes, however I think some of the findings and implications of them should be discussed more clearly.
- Are the limitations of analysis clearly described? The limitations of the presented data should be discussed more clearly.
- Do the authors discuss how these data can be helpful to advance our understanding of the topic under study? Yes, but I suggest that the authors should revise and improve the discussion. There are various interesting findings:
  - In the author summary they state “…its performance varied according to the density of microfilariae in the blood.” And this is also visible in table 4. And how do the authors explain the differences in test performance depending on reference diagnostics? I suggest to better discuss this finding and compare it to available data.
  - In the abstract conclusion the authors state that “…IgG and IgG4 antibody responses should be interpreted differently”. This is an interesting finding, but it is not further discussed. What could be the implications?
- Is public health relevance addressed? I suggest to further elaborate this point in the discussion – do the author’s findings provide new possibilities for loiasis diagnostics or mapping strategies? If not, why? What could be improved in the development of future tests?

**We would like to thank the reviewer for his comments and suggestions, and for his general appreciation of the article.**

**We have added sentences in the discussion in order to address the reviewer comments.**

- **“**This unexpected result warrants further investigation. IgG4 has been identified as the predominant IgG isotype in L. loa infection. Interestingly, anti-Loa IgG4 recognizes not only antigens from adult worms but also those from L3 and microfilarial stages. Notably, there is no correlation between microfilarial densities and elevated levels of anti-Loa IgG4 [15,16].
- To identify high microfilarial densities (> 10,000 mf/mL), the pan-IgG test demonstrates higher sensitivity compared to the IgG4 test. Similarly, when focusing on occult loiasis, the pan-IgG test also shows higher sensitivity than the IgG4 test. This could be due to the presence of another IgG isotype, necessitating further investigations.
- We have also shown that diagnostic performance of both ARTs decrease as the microfilaremia of individuals increases. Veletzky *et al*. also found that individuals with high MFD often tested negative by the pan-IgG ART and ELISA [17]. Several hypotheses can be proposed: (i) the overproduction of mfs could block the antibody reaction mechanically or by direct binding with the antibody, (ii) high microfilaremia may be due to lower levels of immunity, resulting in reduced antibody levels, or (iii) the overproduction of microfilariae could lead to alterations in the spleen, as described in various articles [18–21], causing a reduction in splenic function and, consequently, altered antibody production. These hypotheses require further study.**”.**
- **“**While various strategies have been evaluated to combat onchocerciasis in areas where it is hypo-endemic and co-endemic areas for loiasis, none have been retained yet [17]. This underscores the need for alternative diagnostic tools that can identify individuals at risk of serious side effects. Although IgG4-test do not fully address this issue, it may contribute to the diagnosis and mapping of loiasis. This can help identify areas at risk and offer a cost-effective solution for managing both onchocerciasis and loiasis**”.**
- *Line 34: “Diagnosing loiasis is challenging due to sporadic and non-specific clinical symptoms.” This sentence may be misread, rephrase for clarity.*
- *Line 35 and later: I would call them larvae and not embryos*
- *Line 49: do you mean “experienced”?*
- *Line 63: do you mean “underlines”?*
- *Line 67: do you mean swelling<s> or maybe add more details? And I suggest to rephrase “Treating it is tricky,…”*
- *Line 68: state the type of test that you tested (i.e. rapid diagnostic…)*

**We have made the changes requested by the reviewer.**

*Line 82: 40% seems quite high especially if you are referring to the total population (and not only adults).*

**Indeed, we have replaced this figure by 30% in order to include minors in the prevalence estimate.**

*Line 85-86: add citations for symptoms, the provided citations do not describe these general symptoms.*

**We add 2 citations:**

Ramharter et al. (2023). The African eye worm: current understanding of the epidemiology, clinical disease, and treatment of loiasis. *The Lancet Infectious Diseases*.

Veletzky et al. (2020). Burden of disease in Gabon caused by loiasis: a cross-sectional survey. *The Lancet Infectious Diseases*.

*Lines 94-96: Is it only (host) genetics or is the situation more complex?*

**It is indeed related to the host genetics. Garcia et al. (1999) results indicate that there is a genetic predisposition to be microﬁlaraemic and that predisposed subjects might be genetically unable to mount an efﬁcient immune response against loiasis antigen. However, this topic remains understudied and genetic studies such as GWAS (genome wild association studies) should be done to identify the nature of the genetic factors predisposing to microﬁlaraemia.**

*Line 104-106: rephrase for clarity*

**We rephrase this sentence: “**Current guidelines permit the use of ivermectin-based MDA in areas where *Loa loa* and onchocerciasis coexist, provided that onchocerciasis is meso- or hyperendemic. However, it is essential to establish close surveillance to promptly identify and manage any adverse events following ivermectin administration**”.**

*Lines 112-113: I think “of antibodies” may be removed from the sentence and maybe: ll-SXP-1, a marker of exposure to loa loa infection in the laboratory setting or similar.*

**We removed “of antibodies”.**

*Line 119: remove the second “as possible” and lines 119-123: this phrase is too long, rephrase for clarity.*

**We rephrased this sentence: “**The test was designed to be highly sensitive when read with the naked eye. Additionally, it can be quantified using a smartphone-based chromatographic test reader or a scorecard. This allows users to set a threshold for test line intensity, determining when the test is considered positive. By adjusting this threshold, users can balance sensitivity and specificity according to their needs**”.**

*Line 167: why were the slides dehemoglobinized before staining?*

**Since *Loa loa* mfs are approximately the same size as red blood cells, the numerous red blood cells could hide the mfs. In order to make the mfs clearly visible under microscopy, it is useful to remove the red blood cells by means of a dehemoglonization step.**

*Line 169: how were discordant results handled?*

**We added the following information: “**Slides with an MFD difference exceeding 30% between the 2 readings were reread blind to the first result**”.**

*Line 183: how were eyeworm and calabar swelling queried? Using the raploa questionnaire or similar methods?*

**We used the RAPLOA questionnaire: “**Collected data encompassed demographic information and specific loiasis-related signs: eye worm and transient edema, using the RAPLOA questionnaire**”.**

*Line 201: I think it should say “infection was assessed”*

**We used the pan-IgG test as a reference to evaluate the diagnostic performance of the IgG4 test to detect *L. loa* infection. Therefore, we believe our sentence is acceptable: “**Diagnostic performance of the IgG4 test to detect *L. loa* infection has been assessed using the pan-IgG test as the reference**”.**

*Lines 233-230: As I understand the presentations, the numbers don’t add up. Maybe redefine for clarity.*

**We thank the reviewer for this comment. We made the appropriate corrections: “**Among this group, 21 individuals were microfilaremic, 58 had exhibited at least once a loiasis-related sign, and 12 had both microfilaremia and a history of loiasis-related sign (with 24 cases having missing data)**”.**

- *Line 242-243: in “the” tested population*
- *Line 269: for “the” IgG test…*
- *Lines 330-332: rephrase for clarity*
- *Line 324: sera?*

**We made the requested corrections.**

*This paper by Campillo et al describes a comparison of two different rapid diagnostic tests detecting IgG and IgG4 antibodies against Loa loa. Diagnosis of loiasis remains difficult and data on RDT performance, especially from endemic populations, is limited.*

*The authors clearly describe the used methods and present the data straight forward. However, I think that certain points deserve more attention, should be put into context and the discussion should be expanded (see points below).*

**We appreciate the reviewer feedback and have made modifications to the manuscript discussion to provide a clearer understanding and better context for our results. We hope these changes address the reviewer concerns and enhance the overall discussion.**

_________________________________________________________________________________

**Reviewer #3**.

*No onchocerciasis patient was formally identified, further studies were suggested, and this should be done in onchocerciasis known endemic areas.*

**We agree with reviewer 3. We also believe that it will be of great interest.**

*Test-and-(not)-treat should be well noted Test and not Treat (TaNT)*

**We made the modification.**

*This paper contributes to the evaluation of diagnostic tests for onchocerciasis and loiasis, which are major concerns for the onchocerciasis elimination program. The major concern for the Test and Not Treat (TaNT) strategy is the identification of subjects at risk of Serious Adverse Events (SAEs). The LoaScope exists and is used for this strategy in onchocerciasis hypo-endemic areas and co-endemic areas for loiasis. However, there are constraints associated with this tool. It would therefore be useful to work on other diagnostic tools that can be used to identify subjects at risk of serious side-effects. The diagnostic tools presented in this paper do not solve this problem, but at least contribute to the diagnosis of loiasis, which can contribute to the mapping of loiasis and thus help to identify areas at risk. The fact that these tools can be used for both onchocerciasis and loiasis presents a major advantage in terms of cost.*

**We thank the reviewer for their appreciation of our paper. We acknowledge that our study does not address the problem of identifying subjects at risk of SAEs in co-endemic areas. However, we believe that this initial research on the biplex RDT will spark interest in further studies focused on the diagnosis of loiasis. Our work contributes to the broader goal of mapping loiasis, which can ultimately aid in identifying areas at risk and improving the overall strategy for onchocerciasis and loiasis management.**
